# Role of Helicase-Like Transcription Factor (Hltf) in the G2/M Transition and Apoptosis in Brain

**DOI:** 10.1371/journal.pone.0066799

**Published:** 2013-06-24

**Authors:** Rebecca A. Helmer, Oded Foreman, Janet S. Dertien, Marlyn Panchoo, Suhani M. Bhakta, Beverly S Chilton

**Affiliations:** 1 Department of Cell Biology & Biochemistry, Texas Tech University Health Sciences Center, Lubbock, Texas, United States of America; 2 Genentech, Inc., South San Francisco, California, United States of America; 3 Department of Pharmacology & Neuroscience, Texas Tech University Health Sciences Center, Lubbock, Texas, United States of America; 4 St. George's University, St. George's, Grenada, West Indies; Beijing Institute of Microbiology and Epidemiology, China

## Abstract

HLTF participates in transcription, chromatin remodeling, DNA damage repair, and tumor suppression. Aside from being expressed in mouse brain during embryonic and postnatal development, little is known about Hltf's functional importance. Splice variant quantification of wild-type neonatal (6-8 hour postpartum) brain gave a ratio of 5:1 for Hltf isoform 1 (exons 1-25) to isoform 2 (exons 1-21 with exon 21 extended via a partial intron retention event). Western analysis showed a close correlation between mRNA and protein expression. Complete loss of Hltf caused encephalomalacia with increased apoptosis, and reduced viability. Sixty-four percent of Hltf null mice died, 48% within 12-24 hours of birth. An RNA-Seq snapshot of the neonatal brain transcriptome showed 341 of 20,000 transcripts were altered (p < 0.05) - 95 up regulated and 246 down regulated. MetaCore^TM^ enrichment pathway analysis revealed Hltf regulates cell cycle, cell adhesion, and TGF-beta receptor signaling. Hltf's most important role is in the G2/M transition of the cell cycle (p  =  4.672e-7) with an emphasis on transcript availability of major components in chromosome cohesion and condensation. Hltf null brains have reduced transcript levels for Rad21/Scc1, histone H3.3, Cap-E/Smc2, Cap-G/G2, and Aurora B kinase. The loss of Hltf in its yeast Rad5-like role in DNA damage repair is accompanied by down regulation of Cflar, a critical inhibitor of TNFRSF6-mediated apoptosis, and increased (p<0.0001) active caspase-3, an indicator of intrinsic triggering of apoptosis in null brains. Hltf also regulates Smad7/Bambi/Tgf-beta/Bmp5/Wnt10b signaling in brain. ChIP confirmed Hltf binding to consensus sequences in predicted (promoter Scgb3a1 gene) and previously unidentified (P-element on chromosome 7) targets. This study is the first to provide a comprehensive view of Hltf targets in brain. Moreover, it reveals how silencing Hltf disrupts cell cycle progression, and attenuates DNA damage repair.

## Introduction

HLTF was first described as a DNA-binding protein [1]–[3] and later as a *bona fide* transcription factor [4]–[9]. A SWI/SNF family member cloned and characterized in human [1], [4], [6], mouse [3], [5] and rabbit [2], HLTF is capable of local and long-range regulation. For example, acting through promoter elements, HLTF mediates the ability of prolactin to augment progesterone-dependent transcription of the rabbit uteroglobin (SCGB1A1) gene [7], the founding member of the secretoglobin (SCGB) gene family [10], and transcription of the human beta-globin gene [9]. HLTF utilizes its DNA-looping ability to auto regulate [11]–[13], control blue-brown eye color via Oca2 [14], [15], and mediate circadian prolactin transcription [16].

HLTF is a tumor suppressor [17] silenced by promoter hypermethylation in gastrointestinal tract [18]–[21] and select uterine cancers [22]. Methylation of HLTF’s promoter occurs in the early events of the adenoma-carcinoma sequence [23]. Sandhu et al [24] recently showed Hltf deficiency on the Apc min/+ mutant background increased the formation of intestinal tract tumors with concomitant gross chromosomal instabilities. The requirement for HLTF in error-free postreplication repair of damaged DNA is consistent with its role in cancer-suppression [25], [26].

HLTF is the mammalian ortholog of yeast Rad5. As a mammalian DNA damage response gene, HLTF maintains genome stability by promoting the Lys-63-linked polyubiquitination of proliferating cell nuclear antigen (PCNA) via its ubiquitin ligase activity [27], [28]. HLTF promotes error-free replication of damaged DNA [26], [29], [30], and protein clearing on stalled replication forks [31], [32]. The deubiquitylating enzyme ubiquitin-specific protease 7 (USP7) extends the half-life of HLTF thereby promoting HLTF induced PCNA polyubiquitination [33]. USP7-HLTF-PCNA comprises a newly identified molecular network regulating DNA damage response [33].

Knocking out either USP7 [34] or PCNA [35] results in early developmental lethality in mice. Thus we generated a conditional knockout allele of the Hltf gene by flanking sequences that encode the nuclear localization signal (NLS) with loxP sites, and converted it to a null mutation with a premature termination codon by transmitting the targeted allele through the female germline of cytomegalovirus (CMV)-Cre mice. This strategy provided the flexibility to breed Hltf-floxed mice to other Cre-expressing lines in the event that the constitutive knockout caused developmental lethality. Deleting the NLS coding sequences and introducing a premature stop codon sentenced all transcripts to nonsense-mediated decay (NMD), and no proteins were translated. Because Hltf is a multifunctional protein, the phenotype of the null mouse derives from the absence of transcriptional regulation by Hltf, and the loss of Hltf in DNA damage repair. The brain – with widespread encephalomalacia, increased apoptosis, and defects in the G2/M transition - is one organ of the body profoundly affected by Hltf silencing.

## Materials and Methods

### Reagents and Kits

Santa Cruz Biotechnology, Inc. (Santa Cruz, CA) was the source of Protein A/G PLUS-Agarose (sc-2003) beads, goat anti-mouse IgG-HRP (sc-2005), donkey anti-goat IgG HRP (sc-2033) and mouse brain extract (sc-24879). GE HealthCare (Pittsburgh, PA; formerly Amersham Biosciences) is the source of donkey anti-rabbit IgG HRP (NA934V). Ready Gel Tris-HCl precast polyacrylamide gels (7.5%, #161-1154, and 10%, #1611155), and Immuno-Star Western Kit (170–5070) were purchased from Bio-Rad (Hercules, CA). PerkinElmer Life Science Products (Waltham, MA) was the source of Kodak Film (NEF596). Sudan Black B (199664) was purchased from Sigma-Aldrich (St. Louis, MO). The Caspase 3 Assay Kit (ab39401) was purchased from Abcam (Cambridge, MA).

Antibodies for NeuN (MAB377) were purchased from Millipore (Billerica, MA). Antibodies to HLTF - N-terminus, residues 164–300, and residues 600–700 - were purchased from Santa Cruz Biotechnology, Inc. (sc-27542X), Sigma-Aldrich (HPA015284), and Abcam (ab17984), respectively. Invitrogen Corp. (Carlsbad, CA) was the source of Alexa-conjugated antibodies - Alexa Fluor 488 chicken anti-rabbit IgG (A21441) and Alexa Fluor 594 chicken anti-mouse IgG (A21201) – and prolong gold antifade reagent with 4′,6-diamidino-2-phenylindole (DAPI).

Mouse brain extract was prepared with the Active Motif (Carlsbad, CA) nuclear extract kit (#40010). DNeasy Blood & Tissue Kit (69506) was purchased from Qiagen (Valencia, CA) for isolation of genomic DNA from tail biopsies. Expand Long Template PCR System Buffer (11681842001) and PCR nucleotide mix (11814362001) were purchased from Roche Applied Science (Indianapolis, IN). PCR primers (Table 1) were synthesized by Midland Certified Reagent Company (Midland, TX). OmniPur agarose (2120) was purchased from Calbiochem division of EMD4Biosciences (San Diego, CA), and MetaPhor® agarose (50181) was purchased from Lonza Rockland, Inc. (Rockland, ME). Promega (Madison, WI) was the source of agarose gel markers (G171A, G173A, andG176A).

**Table 1:** PCR Primers.

Name	Sequence
Floxed Forward	5′-ACCTCATCAATTGACATCTTAATCGGTCG-3′
Floxed Reverse	5′-CTGCCAAGAAGATACTCCAAATCTGTTCACTAC-3′
Knockout Forward	5′-GTTAGGAGTGTTCTGCGTTCTAGGACTGATG-3′
Knockout Reverse	5′-AGAAGAATTTTATGGGCTGAAGGTGGG-3′
P element Forward	5′-CCCTGTGCAGCAGAGTAGCTCATTAAC-3′
P element Reverse	5′-GGAAAAACAGAGCCAAATGATCCCAGGCTTG-3′
Scgb3a1 Forward	5′-GCTCTCAGACCAGCAGGAGCTGAG-3′
Scgb3a1 Reverse	5′-CTCTGTGTGGCTCTGCTCAGTGACTC-3′

For RNA-seq, Otogenetics Corporation (Norcross, GA) used the following reagents: Ribo-Zero rRNA Removal Kit from Epicentre (Madison, WI), an Illumina company; NEBNext mRNA Sample Prep kit (E6110) and NEBNext reagents (E6040) from New England Biolabs (Ipswich, MA).

### Techniques

Microscopy, chromatin immunoprecipitation (ChIP), and Western analyses were performed as previously detailed [7], [12], [36], [37]. For histological evaluation, heads were severed from torsos of newborn mice at 6–8 hours of age. Heads and torsos were emersion-fixed in a variety of formalin-based fixatives. For torsos, a long incision was made in the abdominal and pleural wall along the midline to allow fixative penetration. Each head was paraffin embedded in a block in coronal orientation and each torso in another block at a sagittal orientation. Blocks were serially sectioned (5–8 mm) at 250 micron intervals. Hematoxylin and eosin (H&E) stained sections were evaluated by light microscopy.

### Hltf Null Mice

Hltf knockout mice were developed in collaboration with genOway (Lyon, France). All studies were conducted in accord with the NIH Guidelines for the Care and Use of Laboratory Animals, as reviewed and approved by the Animal Care and Use Committee at Texas Tech University Health Sciences Center [NIH Assurance of Compliance A3056-01; USDA Certification 74-R-0050, Customer 1481]. TTUHSC’s IACUC specifically approved this study. All efforts were made to minimize pain and suffering.

The *Hltf* locus was analyzed approximately 8.5-kb on each side of exon 1 for GC content, repeats and secondary structure. For promoter analysis, web-based tools (ConSite and ConReal) were used for finding putative *cis*-regulatory elements in genomic sequences. Predictions were based on the integration of binding site predictions generated with high-quality transcription factor models, and cross-species comparison filtering (phylogenic footprinting). Consensus (K-K/R-X-K/R) nuclear localization sequence (NLS) based on *in silico* analysis with PredictNLS through the PredictProtein server was used to identify a single monopartite NLS at position 380 (VCPKRRKISV; score 7) encoded by exons 10–11. Primo-analysis was used to lower the probability of random PCR priming by comparing primer sequences with the mouse transcriptome.

**Figure 1:**
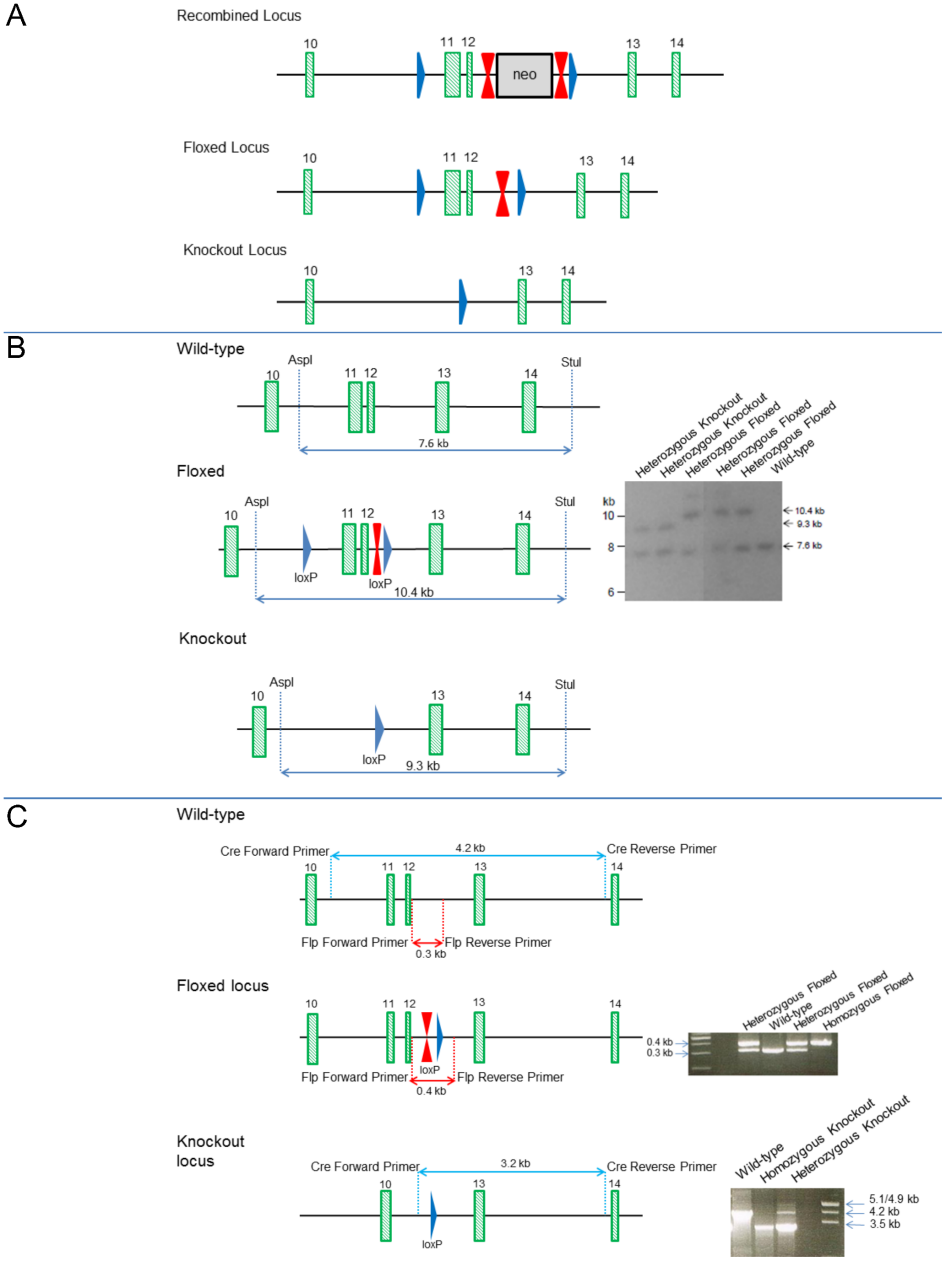
Loci from Hltf floxed and constitutive knockout mice. Panel A, recombined allele in F1 mice, i.e. FRT-flanked (double red triangles) Neo resistance selection cassette is downstream of exon 12, and two loxP sites (blue triangles) are upstream of exon 11 and downstream of exon 12. Exons are numbered green rectangles. Next, the floxed allele in F2 mice results from breeding F1 mice with Flp recombinase-expressing C57BL/6J Flp deleter mice to remove the Neo resistance cassette. Lastly, the knockout allele (F3 mice) results from breeding F2 heterozygous Neo-excised Hltf-floxed mice to C57BL/6J mice expressing Cre recombinase under the control of the CMV promoter. Panel B, schematic of the Hltf wild-type, floxed, and knockout alleles with restriction sites for the 3′-Southern blot strategy. Southern analysis was performed with DNA digested with Asp1-Stu1, blotted on nylon membrane, and hybridized with the 3′- probe detecting the Asp1-Stu1-fragments of expected sizes (kb). Panel C, schematic of genotyping by PCR shows wild-type, floxed and knockout alleles with primer locations, and expected amplicon size. PCR amplicons for each genotype are provided with DNA size markers.

**Figure 2:**
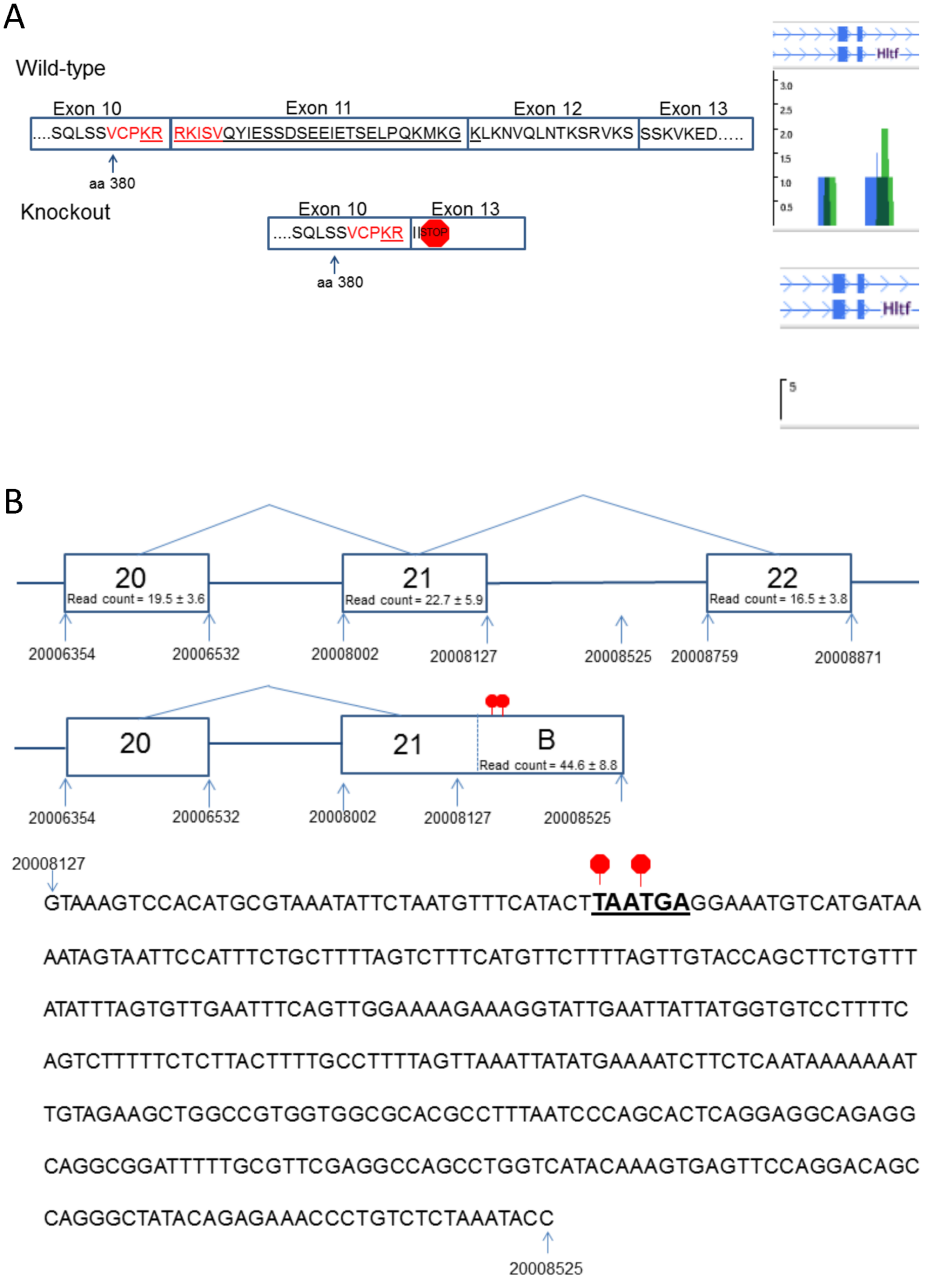
Splicing events in Hltf. Panel A, diagram showing the presence (wild-type) and absence (Hltf null) of sequences encoding the NLS, and a DNAnexus genome browser screen shot of RNA-seq data verifying deletion of the sequences from Hltf null transcripts. Panel B, illustrates partial intron inclusion event (B) that produces Hltf isoform 2, which ends abruptly in dual stop codons.

Sequences encoding the NLS were targeted by inserting a FRT-neomycin-FRT-LoxP selection cassette downstream of exon 12, and a LoxP site upstream of exon 11 (Figure 1A). After homologous recombination (129Sv/Pas ES cells), ES cell injection, and generation of germline chimeras, chimeric mice were bred to C57BL/6J mice to generate F1 mice carrying the recombined floxed Hltf allele and Neo selection cassette. The Neo cassette was deleted by breeding F1 mice with Flp recombinase-expressing C57BL/6J mice. Heterozygous neo-excised Hltf-floxed mice were bred to C57BL/6J mice expressing Cre recombinase under the direction of the CMV promoter. This strategy caused 125-bp of sequence encoding the NLS to be deleted by Cre recombinase in the early stages of embryonic development. Rejoining of exon 10 and exon 13 produced a frame shift that introduced a premature termination codon (PTC) in exon 13 (Figure 2A). Heterozygous Hltf-deficient mice (F3 generation of C57BL/6J backcross) were used in the initial characterization of the Hltf null phenotype while the Hltf-deficient mouse strain was backcrossed into the C57BL/6J genomic background for 10 generations.

### Genotyping

PCR screening reactions (Figure 1C) were used for the detection of amplicons unique to the Hltf floxed allele (329-bp wild-type, 329/424-bp heterozygous, 424-bp floxed), and the Hltf knockout allele (4195-bp wild-type, 4195/3224-bp heterozygous, 3224-bp homozygous knockout). Each 50 µl PCR reaction consisted of genomic DNA (60 ng), primer pairs (15 pmol each, Table 1), dNTPs (0.5 mM), reaction buffer 3 (0.1 Vol), and expand long template polymerase (2.6 U). Reaction conditions for the floxed allele were as follows: 120 sec at 94 C, followed by 35 cycles of 94 C for 30 sec, 65 C for 30 sec, and 68 C for 120 sec, and a final extension for 480 sec at 68 C. Reaction conditions for the knockout allele were identical to those for the floxed allele except extension at 68 C was increased to 240 sec. At the conclusion of each reaction, samples were rapidly cooled to 4 C, and amplicons were resolved/visualized by MetaPhor® agarose (floxed allele) and omnipur agarose (knockout allele) gel electrophoresis.

### RNA-seq

Individual samples [2 brains/sample × 3 biological replicates for test and control mice  = 6 total samples] were flash frozen and sent to Otogenetics Corp. (Norcross, GA) for RNA-seq assays. Total RNA was isolated; its integrity and purity were assessed using Agilent Bioanalyzer and OD260/280 (Table 2). Each sample was rRNA-depleted using the appropriate Ribo-Zero rRNA Removal Kit according to the manufacturer. cDNA was generated using NEBNext mRNA Sample Prep kit from the rRNA-depleted RNA. cDNA was profiled using Agilent Bioanalyzer, and subjected to Illumina library preparation using NEBNext reagents. The quality, quantity and size distribution of the Illumina libraries were determined using an Agilent Bioanalyzer 2100. The libraries were then submitted for Illumina HiSeq2000 sequencing according to standard operation. Paired-end 100 nucleotide reads were aligned to genomic assembly mm9 (Table 2) and analyzed using the platform provided by DNAnexus, Inc. (Mountain View, CA).

**Table 2:** Sample quality control and RNA-seq outcome.

Sample ID	OD260/280	RIN†	Total Bases	TotalReads	Mapped Reads
1-Control	2.1	8.4	2,373,285,000	23,732,850	79.68%
2-Control	2.12	7.5	2,440,788,800	24,407,888	81.19%
3-Control	2.06	7.5	2,473,098,200	24,730,982	82.12%
4-Null	2.11	8.2	2,884,347,200	28,843,472	81.47%
5-Null	2.10	8.6	2,483,495,000	24,834.950	53.10%
6-Null	2.10	8.0	2,302,117,800	23,021,178	81.46%

† High RNA integrity number (RIN) scores (7–10) and a narrow distribution of scores.

(1–1.5) from an Agilent Bioanalyzer indicated high RNA sample quality.

### Data Analysis

RNA-seq in conjunction with 3SEQ/transcriptome was used to quantify expression levels by comparing sequencing reads against a reference transcriptome. Each transcript was quantified by calculating its RPKM (reads per kilobase of transcript per million mapped reads) enabling direct comparisons of expression levels among transcripts and across experimental conditions. RPKM and total read counts were reported for each gene.

Otogenetics via DNAnexus provided an unbiased gene expression analysis report of RNA-seq; alternative splicing analysis of Hltf; mutation/RNA-editing analysis and parallel comparison of expression profiles between null and control samples. FPKM (fragments per kilobase of transcript per million mapped reads) were mapped against mm9 with Tophat (V1.3.3) to obtain.bam mapping files that were input into Cuffdiff (V 1.3.0) for comparison between two conditions (null, control) and three replicates for each condition. Using a free trial, data were imported to MetaCore™ for pathway analysis (GeneGO, Thomson Reuters, New York, NY). Standard enrichment parameters (1.1, p<0.05) were used.

Caspase 3 assays were performed on whole brain extract according to the manufacturer’s protocol. Triplicate values were evaluated by ANCOVA (*p*<0.05 significance level) using GraphPad Prism V.6.0b (GraphPad Software). The *p* value for the difference between elevations caused by the presence or absence of Hltf expression was *p*  = 0.0003. Regression analyses of expression level measured by RPKM for control vs. Hltf null values were achieved with GraphPad Prism V.6.0b.

## Results

Mouse *Hltf* is located on chromosome 3qA3 and extends over 60.5 kb. The cDNA sequence (NM_009210) was used to establish the 25-exon/24-intron organization of the Hltf gene. To generate an Hltf knockout mouse model, three different targeting strategies were attempted: 1) exon 1 to eliminate the ATG; 2) exons 4–6 to eliminate the HIRAN (HIP116, Rad5p N-terminal [38]) domain and part of the DNA-binding domain; and 3) exons 11–12 to eliminate the NLS coding sequence. Secondary structure, repetitive sequences, and large regions with GC content below 30% compromised the first two strategies. Thus, the third strategy was selected (Figure 1A).

**Figure 3:**
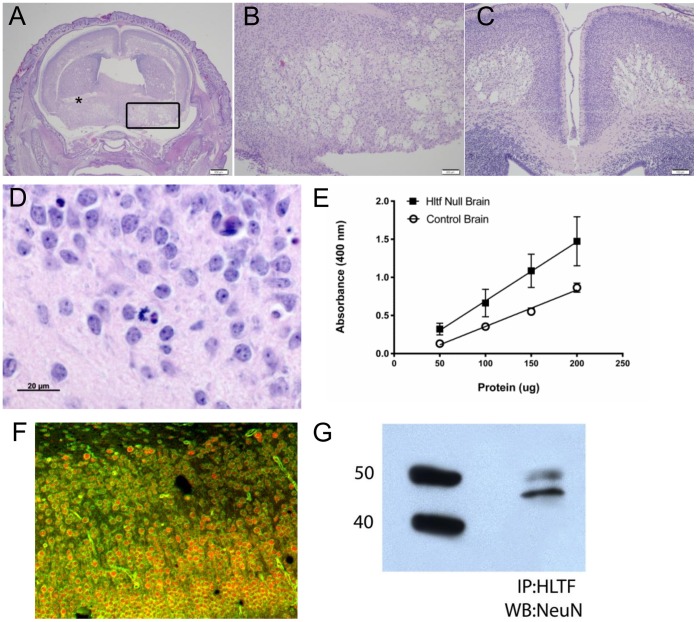
Hltf expression in null and control brains. H&E stained coronal sections from newborn Hltf null mouse brains (A–D); caspase 3 assays comparing null and control brains (E); immunofluorescence image (F) and Western analysis of immunoprecipitates (G) from control brains. Panel A, widespread vacuolation in the anteriorcommissure (*) and preoptic/ventral paladium (boxed region). Panel B, higher magnification of the cholinergic-rich ventral paladium involved in regulation of motivation and behavior. Panel C, extensive bilateral vacuolation in the motor cortex. Panel D, apoptotic cells (pyknotic fragmented nuclei, condensed and hypereosinophilic cytoplasm) in regions of encephalomalacia. Panels E, active caspase-3 shows increased intrinsic triggering of apoptosis in Hltf null brains. Panel F, acquired confocal images were imported into Metamorph 6.3 image analysis software and merged to show NeuN (red) and Hltf (green) colocalize in nuclei (orange) of neurons in the motor cortex. Panel G, NeuN/Fox-3, a member of the Fox-1 gene family of splicing factors, and marker for nuclear speckles, coimmunoprecipitates with Hltf.

**Figure 4:**
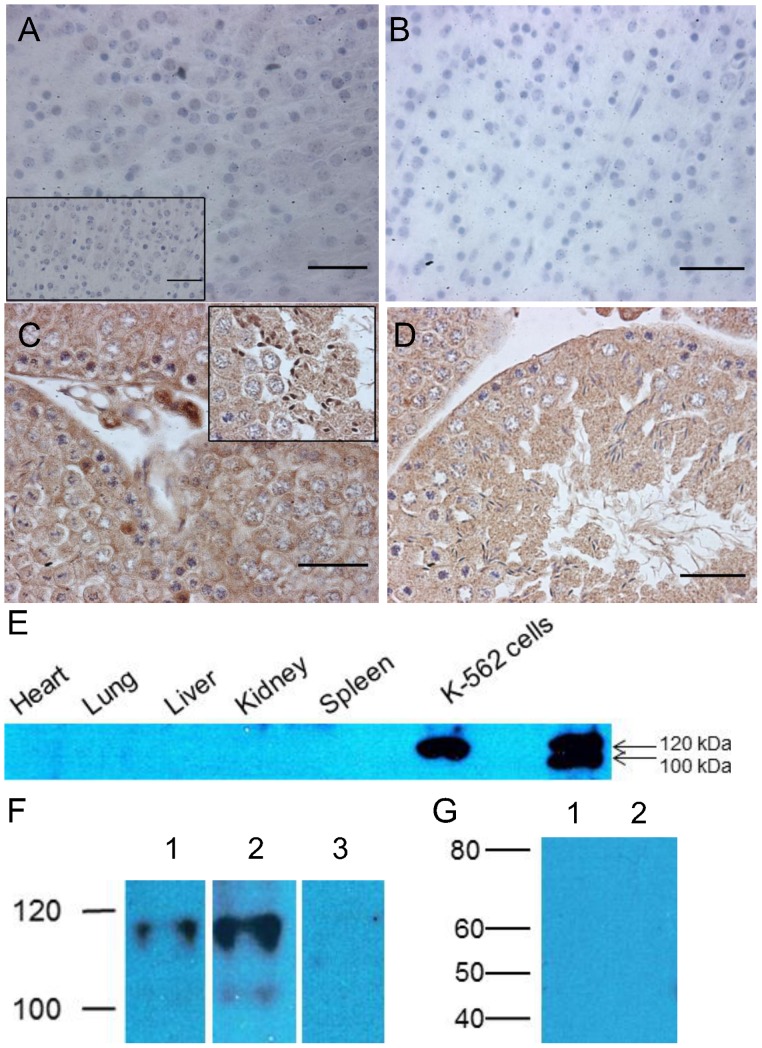
Hltf expression in null and control tissues. Panel A, immunostained coronal section from motor cortex of newborn control brain at 40X with a 20X (inset) shows pale staining, compared with the no antibody control at 40X in Panel B. Panel C, comparatively strong immunostaining in what appear to be secondary spermatocytes and leydig cells (40X) was well as spermatids (40X inset) in seminiferous tubules from newborn control mice. Panel D, Hltf immunostaining was absent from all stages of spermatogenesis in Hltf null seminiferous tubules (40X). Panel E, Western blot of immunoprecipitates shows the absence of Hltf protein from tissues from Hltf null mice compared with K-562 cells (robust, positive control for 115-kDa human Hltf). Panel F, Western blots of Hltf expression in brain extracts of wild-type control mice (lanes 1 and 2), and brain extracts of null mice (lane 3). Hltf from full-length Hltf mRNA isoform 1 is shown in lane 1. Prolonged exposure (10 minutes) of Western blots from control brain (lane 2) revealed faint evidence of a protein product from the truncated Hltf mRNA isoform 2. No Hltf is detected in the brains of Hltf null mice by Western blot regardless of the exposure time. Panel G, no truncated Hltf proteins are detected in the brains of Hltf null mice. Antibodies to HLTF - N-terminus (panels C, D and F), residues 164–300 (panel G1), and residues 600–700 (panels A, B, E and G2) – were used. The antibodies used in panel G1 would have identified truncated Hltf protein (44 kDa) from the original ATG, and antibodies used in panel G2 would have identified truncated Hltf proteins (45 kDa, 64 kDa) from the ATG introduced by the deletion of the NLS encoding sequences. Scale bar, 100 µm.

Successful removal of sequences encoding the NLS was confirmed by Southern blot (Figure 1B), PCR analyses (Figure 1C), and RNA-seq (Figure 2A). Hltf deletion caused neonatal lethality, i.e. 64% of all null mice die, 48% during the first 12–24 hours after birth (n = 1527). Histological examination of brains revealed widespread, bilateral spongiform vacuolation (Figure 3A–C) with morphological (Figure 3D) and biochemical (Figure 3E) evidence of increased apoptosis. The first indication that the Hltf null brain phenotype affects neurons was demonstrated by colocalization (Figure 3F) and co-immunoprecipitation (Figure 3G) of Hltf with NeuN/Fox-3 protein, an intrinsic component of neuronal nuclear matrix, in control brain. Faint Hltf immunolabeling was abundant in cells in neonatal wild type brain (Figure 4A) compared with no antibody control (Figure 4B). Immunopositive Hltf cells are found in other tissues such as seminiferous tubules of testes from wild type controls (Figure 4C). By comparison, no immunopositive cells are found in the seminiferous tubules of testes from Hltf null mice (Figure 4D).

RNA-seq confirmed brains from Hltf null mice lacked mutant Hltf transcripts encoding an NLS (Figure 2A). The introduction of a PTC in exon 13 left at least 9 splice junctions between the STOP codon and the polyA signal. The likely hood that NMD would target Hltf mRNA for degradation, and thus prevent translation, was realized when immunoprecipitation plus Western blotting (Figure 4E) confirmed the absence of Hltf in tissues from null mice. Hltf protein (115 kDa) from full-length Hltf isoform 1 is detectable in brain from wild type controls (Figure 4F, lane 1). Increased exposure of the Western blot revealed the smaller Hltf protein (97 kDa) from the truncated Hltf isoform 2 in control brain (Figure 4F, lane 2). No Hltf protein was detected in null brain (Figure 4F, lane 3). Moreover, in Hltf null brain no truncated protein (44 kDa) was translated from the original ATG in exon one (Figure 4G, lane1), and no truncated proteins (45 kDa, 64 kDa) were translated from the new ATG in exon 14 that resulted from deletion of sequences encoding the NLS (Figure 4G, lane 2).

DNAnexus alternative splicing analysis quantified the usage of each exon and each possible splice junction for Hltf in RNA-seq samples from Hltf control brains (Table S1). Hltf isoform 1, the full-length splice variant (4955-bp), contains exons 1–25. Hltf isoform 2, the truncated splice variant (3059-bp), is comprised of exons 1–21 with exon 21 extended via a partial intron retention event (Figure 2B). The resultant mRNA harbors tandem-in-frame premature termination codons. Quantification of isoform expression by Isoform FPKM tracking shows the full-length isoform occurs 5-times more frequently than the truncated splice variant. Based on junction read counts, all additional splicing events are exon-skip events (Table S2). Most of these low frequency events occur only once per sample, and they are not found in all samples.

**Figure 5:**
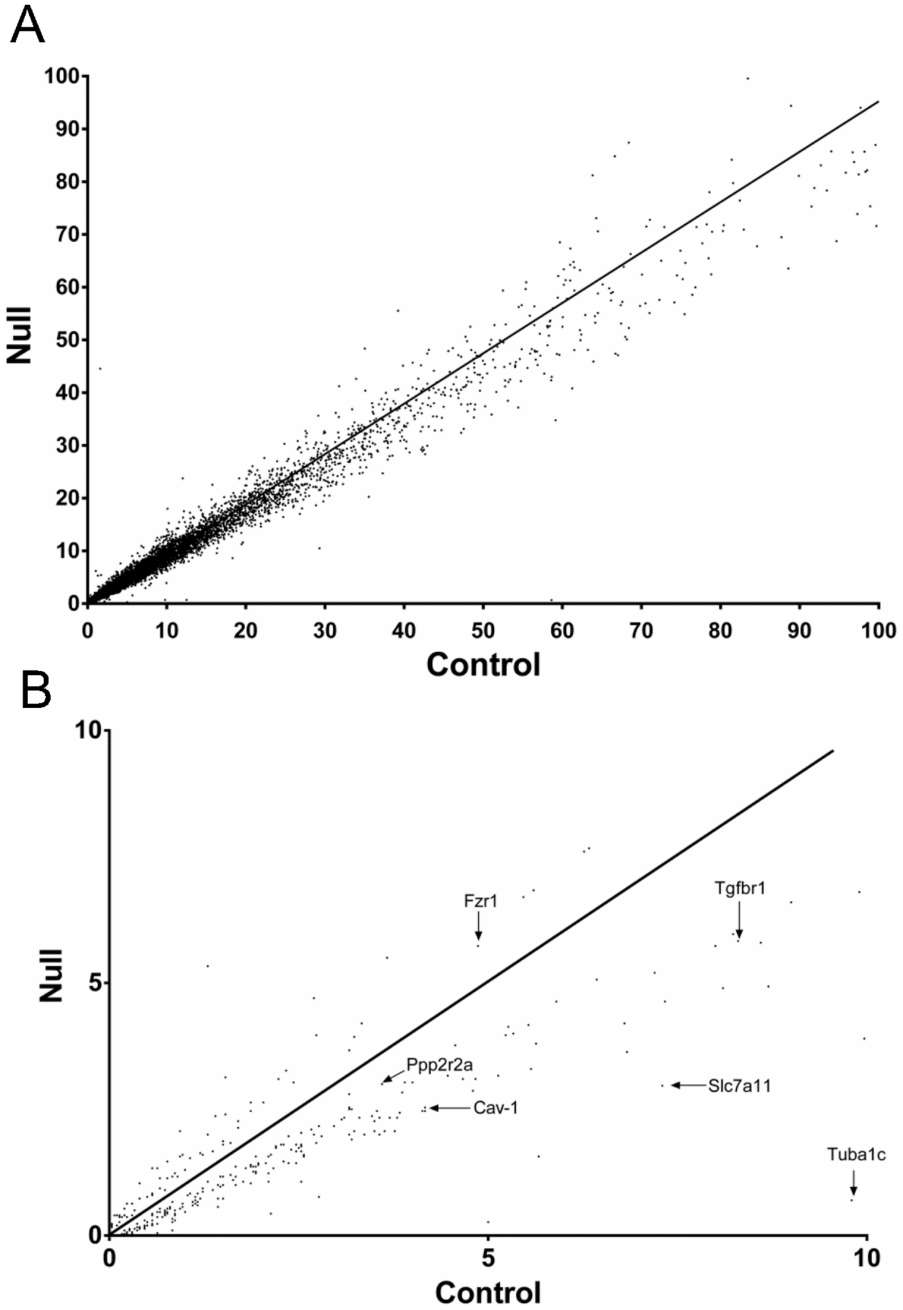
Scatter plot of expression level measured by RPKM: control vs. Hltf knockout. Panel A, each point is the mean of three replicate RPKM values for an individual gene in control brain plotted against values of the corresponding gene in Hltf null brain. The black line indicates the linear regression. R^2^ = 0.9866, correlation coefficient. Panel B, genes with statistically significant expression (each dot is the mean of three replicate RPKM values, differential expression >1.1 fold, and P<0.05 in control vs null brain samples. Genes with dramatic outlier expression such as Tuba1c (14-fold) are indicated.

**Figure 6:**
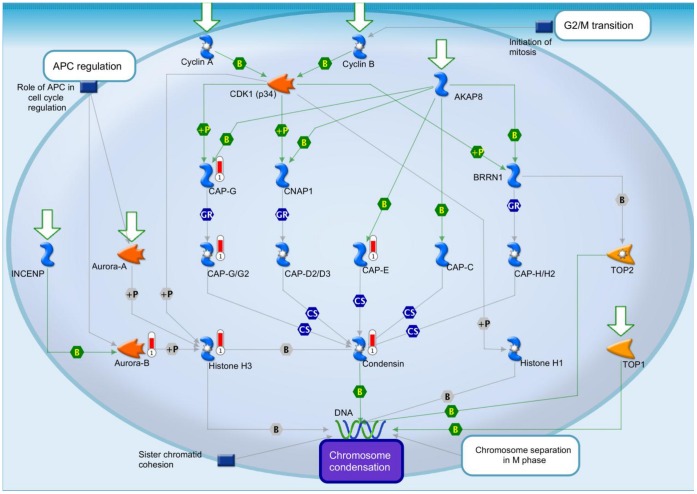
The G2/M transition is the major signaling pathway altered by Hltf deletion. Significant decreases (red thermometers = negative effects) in transcript expression of major components of the G2/M transition in Hltf null brain are superimposed on the proprietary multicomponent canonical pathway map from the MetaCore™ database [Straight lines = interactions; Symbols = events; +P = phosphorylation; B = binding; GR = group relation; CS = complex subunit. Colors for lines and symbols are green for positive, and gray for unspecified].

**Figure 7:**
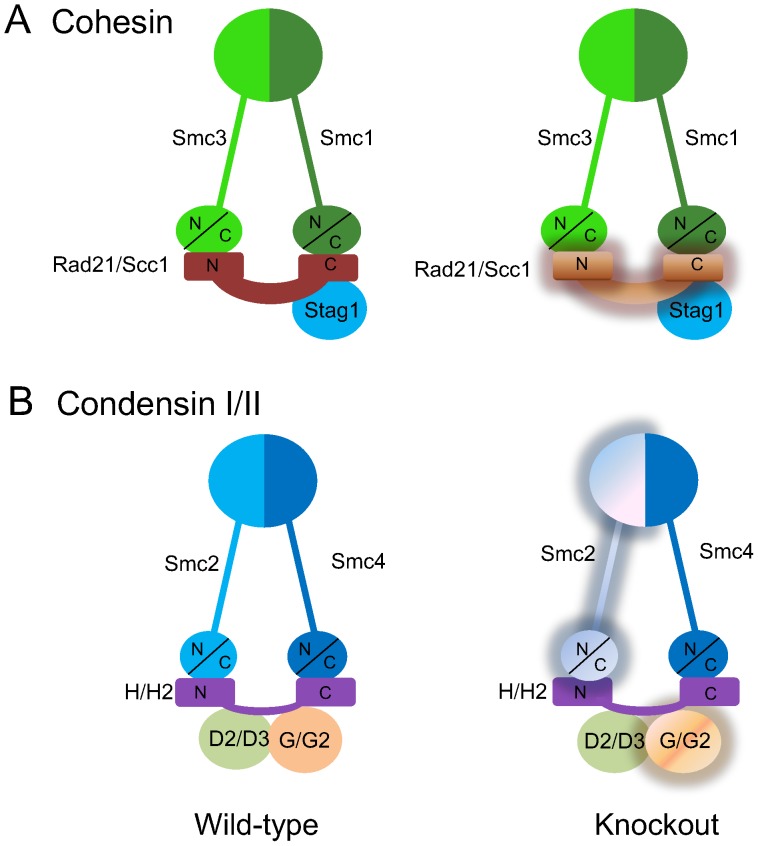
Cohesin/condensin contain structural maintenance of chromosomes (Smc) family of chromosomal ATPases, and non-Smc participants. In Hltf null brain, the functional architecture of each complex is jeopardized by reduced availability (pale shading) of transcripts for major protein components. Panel A, Two twisted Smc proteins (Smc1 and Smc3) that generate V-shaped heterodimers via their hinge domains comprise the core of cohesin. The association of the N- and C-termini of Smc1 and Smc3 with Rad21/Scc1, a kleisin family member that controls separation of sister-chromatids, completes the tripartite ring-like structure. Once properly placed, Rad21/Scc1 is joined at its C-terminus by Stag1 (Scc3). Panel B, Smc2 and Smc4 form V-shaped heterodimers via their hinge domains, and Cap-H/H2, kleisin family members, completes the tripartite ring-like structure. In addition to Cap-H, Condensin I has two additional non-Smc subunits (Cap-D2 and Cap-G). In addition to Cap-H2, Condensin II has Cap-D3 and Cap-G2.

Comprehensive analysis of the brain transcriptome (Figure 5A and Tables S3 and S4) showed 341 of 20,000 total transcripts were altered (p<0.05) - 95 up regulated and 246 down regulated - in Hltf null brains (Figure 5B). MetaCore™ enrichment pathway analysis (Table 3) revealed Hltf is important in the regulation of cell cycle, cell adhesion, and TGF-beta receptor signaling. Hltf’s most important role is in the G2/M transition of the cell cycle (Figure 6) with an emphasis on transcript availability of major components in chromosome cohesion and condensation (p  = 4.672e–7). Cross-talk between structural components of chromosomes is fundamental to the two mechanisms – cohesion and condensation – that govern successful mitosis [39]–[41]. Required proteins are type II topoisomerase, the structural maintenance of chromosomes (Smc) family-members, and non-Smc participants (Figure 7).

**Table 3:** MetaCore™ enrichment pathway analysis.

Pathway	Category	pValue
Cell Cycle	G2/M	4.158E–07
	G1/S	2.982E–03
Adhesion	Gap Junctions	7.375E–05
	Tight Junctions	2.551E–03
Signaling	Activin A	1.982E–03
Development	TGFβ induction of EMT	5.137E–03
	TGFβ receptor signaling	6.491E–03

Transcript levels of topoisomerase (Top IIa and Top IIb) are unaffected (p>0.05) by deletion of Hltf. However, transcript availability of Rad21/Scc1– that encodes one of two major cohesin subunits (Figure 7A; Table S3) that hold sister chromatids together and coordinate chromosome segregation - is dramatically reduced in Hltf null brain. Reduced mRNA levels for Smc2/Cap-E that encodes the structural maintenance chromosome (Smc) subunit common to condensins I and II (Figure 7B), in conjunction with reduced transcript levels of the non-Smc subunits Cap-G and Cap-G2 characterized the Hltf null brain. Transcript levels for Histone H3 are reduced in conjunction with reduced transcript availability of the Aurora B kinase whose protein product phosphorylates Histone H3 protein. Reduced transcript availability for Histone H3 is accompanied by depletion of transcripts for Rad52, the loss of whose protein product promotes cell cycle arrest in G2/M [42].

The loss of Hltf in its yeast Rad5-like role in DNA damage repair - characterized by increased apoptosis (Figure 3D, 3E) – is accompanied by decreased transcript availability for caveolin-1, Bambi, and Smad7 (Table S3), with concomitant reduced transcripts for Cflar (inhibitor of Tnfrsf6-mediated apoptosis [43]). Reduced transcript availability for the TGF-beta type I receptor, which likely results in compromised TGF-beta signaling, is accompanied by a decrease in transcripts for Wnt10b (axonal guidance [44], [45]) coupled with increased (p = 0.039) transcript availability for Gas2L1 (expressed at high levels in growth arrested cells). The Hltf null brain phenotype is further characterized by decreased transcripts (Table S3) for Ska2 mRNA (mitotic arrest [46]); Tnik (neuritogenesis [47]); and Bmp5 and Bambi (neural development [48]).

**Figure 8:**
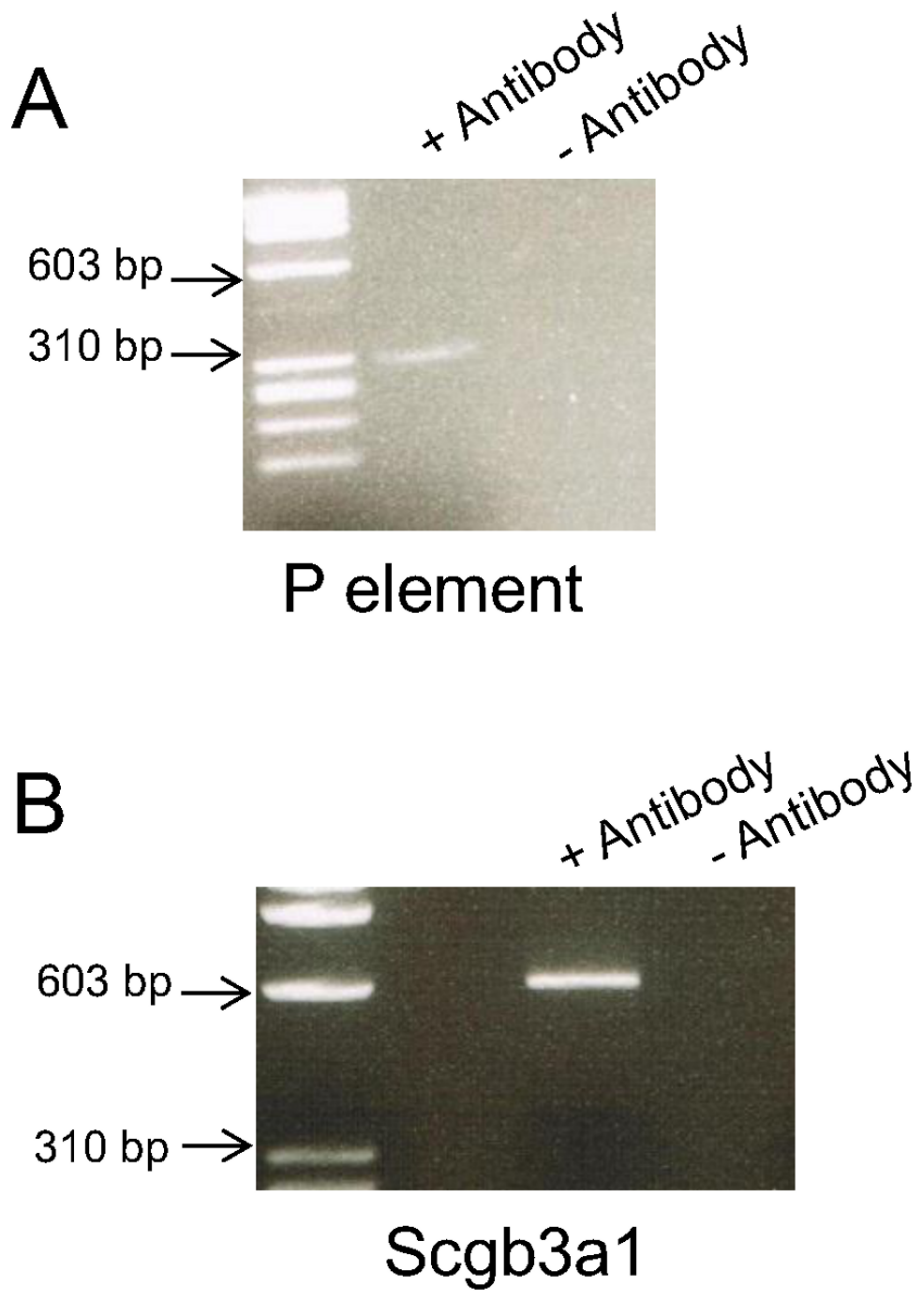
PCR amplification of DNA fragments from ChIP authenticated Hltf binding sites. Panel A, Hltf binds to the ACTTTT sequence in the P-element located between Olfr 713 and Olfr 714 on chromosome 7 [49]. The P element regulates odorant receptor choice. Panel B, Hltf binds the CCATAG sequence in the promoter of the Scgb3a1 gene. The promoter sequence was obtained from the Eukaryotic Promoter Database EPD [50]. Controls (-antibody) and molecular size markers (bp) are provided for each experiment. Amplicons were subcloned and sequenced [7]. The optimal consensus binding sequence [7] for Hltf is C/A C T/A T A/T/G T/G. Genomatics (Munich, Germany) used our data [7] to generate a weight matrix available in MatInspector [51]. Others [13]–[15] used that matrix to identify putative Hltf binding sites in other gene targets.

Newborn Hltf null mice and their littermate controls display a suckling reflex and drink immediately after birth. However, two of three nulls do not survive. This finding correlates with the fact that two out of three nulls are hypoglycemic (data not shown). Hypoglycemia coincides with altered mRNA levels for solute carriers (Slc) that are normally enriched in the blood brain barrier (Tables S3, S4), and upregulation of the brain’s glucose import capacity (Table S4). Although Hltf is not required for the first act of instinctive suckling behavior, altered taste/nutrient detection (Tables S3, S4) potentially modifies subsequent feeding behavior. Thus ChIP was used to authenticate binding of native Hltf to the P-element in chromosome 7 (Figure 8A) that regulates odorant receptor choice [49]. Of equal importance to the characterization of the null brain phenotype is the identification of changes in mRNA levels of known Hltf target genes (Table S3), such as Hbb-b1 [9] or relatives (Scgb3a1; Table S3) of known targets, as well as the correlation of the phenotype with changes in transcripts for key members of E3 ubiquitin ligase pathways (Tables S3, S4) in brain. As shown in Figure 8B, ChIP was used to authenticate an Hltf binding site in the promoter of the Scgb3a1 gene, a member of the SCGB gene family [10].

## Discussion

The strategy for the development of the Hltf null mouse was developed in collaboration with genOway, and utilized the NMD mRNA quality control mechanism [52], [53] to selectively degrade all Hltf messages. The NMD complex recognizes PTCs located at least 50 nucleotides upstream of an exon-intron boundary and selectively degrades them prior to translation. This strategy was successful, thus there are no truncated proteins from the original ATG that would have contained the DNA-binding domain, the HIRAN domain [38], and the WW domain [54]. Also, here are no truncated proteins from the new methionine in exon 13 after deletion of exons 11–12 that would have contained C-terminal original regions of Hltf. All commercially available antibodies to various Hltf residues were utilized in these validation experiments.

The phenotype of our Hltf null mouse differs from a model [24] in which a nuclear localized LacZ c-DNA in frame with the endogenous Hltf start codon replaced exons 1–5 (residues 2–208). LacZ activity in heterozygous mice allowed visualization of Hltf expression throughout development. However, Hltf null mice had no pathologies associated with genetic instability. When the authors knocked down Hltf (shRNA) colon tumor (HCT116) cells, they found evidence of chromosomal instability. Their microarray analysis identified synaptonemal complex protein 3 (Sycp3) as a downregulated (2.39692-fold) target. RNA-seq from Hltf null brain (Table 3S) showed this gene is downregulated (3.125-fold).

HLTF and SHPRH (SNF2 histone-linker PHD-finger RING-finger helicase) are the two human Rad5-related proteins [26]. Like yeast Rad5, they convert monoubiquitinated PCNA into polyubiquitinated PCNA *in vitro*, and cellular loss of either protein increases the frequency of chromosomal abnormalities following DNA damage. In addition to its ubiquitin ligase activity for PCNA, HLTF displays protein-clearing activity at the stalled replication fork. Results from RNA-seq analysis of brains in newborn Hltf null mice indicate the loss of Hltf compromises the DNA-damage avoidance pathway in mouse brain, and modulates mutagenesis by regulating key participants in the G2/M phase of the cell cycle, some of which contribute to DNA double-strand break repair.

Cell division consists of duplication of the DNA content of chromosomes followed by segregation of the chromosomes into daughter cells [41], [42], [46]. Because cohesin mediates cohesion between sister chromatids, it is essential for segregation of chromosomes. Cohesin is normally composed of Smc1, Smc3, Rad21/Scc1, a regulatory subunit that connects them, and Stag1 (Scc3), which affiliates with the C-terminus of Rad21 (Figure 7A). In the Hltf null brain, reduced mRNA expression of Rad21/Scc1 is expected to disrupt the cohesin ring and cause asynchronous anaphase [55]. Also, reduced Rad21/Scc1 mRNA has the potential to compromise the availability of cohesin in DNA looping to regulate transcription [56].

The negative impact of Hltf deletion on cell cycle is not limited to cohesin. It extends to condensin’s role in the compaction of sister chromatids. The condensin complex consists of the two Smc subunits Smc2 and Smc4, and three non-Smc subunits (Figure 7B). Knockdown of Smc2 severely disturbs chromosome condensation [57], and knockdown of Cap-G/G2 promotes aberrant separation of sister chromatids at metaphase [58]. The availability of transcripts for each of these essential components is reduced in Hltf null brain in conjunction with reduced transcripts for the chromosome passenger kinase Aurora B. As such, the Aurora B protein regulates centrosome separation, chromosome segregation, and cytokinesis [59]–[61].

The non-Smc subunits of condensin I and Histone H3 are Aurora B substrates. Aurora B globally phosphorylates Histone H3 on Ser10, and colocalizes with the phosphorylated isoform (H3Ser10ph), which is required for chromosome condensation and segregation. Abrogated expression (RNAi) of Aurora B produced a concomitant reduction in global phosphorylation of Histone H3 [62]. Additionally, targeting Aurora B-mediated H3Ser10ph to repressed genes, as a means of epigenetic silencing gene expression [63], will be decreased in Hltf null brain. In the same Hltf null brain, the reduction in transcript availability for Histone H3 is expected to limit its functional availability as a place holder for the H3 variant, CENP-A another Aurora B substrate [64]. Moreover, reduced transcript availability for Cap-G/G2 is expected to limit the amount of protein available for phosphorylation by Cdc2. These findings add a new Hltf regulatory component to the ability of Aurora B and Cdc2 to govern condensin-chromosome binding during mitosis [65].

Loss of Hltf expression results in apoptosis (Figure 3D, 3E) rather than mitotic catastrophe, an oncosuppressive mechanism [66], tells us something of the molecular profile of Hltf null cells. Analysis of the transcriptome showed ATM kinase-, acinus-, and p53-mediated pathways for cellular rescue/survival vs. apoptosis are intact. However, the activation status of individual protein participants is unknown. Hltf silencing results in the loss of its yeast Rad5-like role in replication of damaged DNA, and the concomitant downregulation of transcript availability for Rad52. Rad5 and Rad52 promote two alternative pathways in postreplication repair (PRR). Because the Rad5 pathway is utilized when the lesion is located on the leading strand template, and the Rad52 pathway is required when the lesion is located on the lagging strand template, we conclude Hltf is required for effective PRR in response to DNA damage that occurs as part of normal brain activity. The next step in providing a molecular definition Hltf’s role in DNA damage repair will be addressed in *in vivo* models that will include the experimental manipulation of the uterine environment.

Hltf/Bmp5 signaling establishes a link between brain development *in utero* and later stages of neural development into adulthood. Low-level Hltf expression was detected by *in situ* hybridization from E11.5-E18.5 [3]. Thereafter, transcripts accumulated in brain. The expression pattern of Bmp5 parallels that of Hltf, i.e. maximal expression on E18 through PN1 [67]. Hltf’s regulation of the Bmp5 morphogen may regulate dendritic morphology [68].

It is clear deletion of Hltf alters the extracellular environment of the brain and as well as its chemosensing ability. For example, reduced (p<0.011) mRNA for Slc7a11, a gene that encodes the cysteine/L-glutamine exchanger xCT, would result in loss of neuroprotection due to reduced cellular glutathione export [69]. Increased (p<0.047) transcript levels for Slc2A9, the gene that encodes Glut9, a sugar-transport facilitator and high capacity/low-affinity urate transporter, potentially alters neurovascular permeability. Hltf also regulates mRNA levels for select olfactory and taste receptors. More importantly, Hltf binds to an authentic site in the P-element in chromosome 7. Since targeted deletion of the P-element in chromosome 7 regulates odorant receptor choice across a cluster of odorant receptor genes [49], loss of Hltf binding to the highly conserved ACTTTT sequence in the P-element might have a similar function. Ectopic expression of odorant receptors [70], especially in testis and sperm, coupled with stage-specific expression of Hltf supports the speculation that Hltf plays a regulatory role in sperm chemotaxis.

In conclusion, the complete loss of Hltf gene product (mRNA, protein) means all genetic and epigenetic functions are lost at the molecular level from cells of Hltf null mice. Beginning with Hltf null brain, because of its compelling morphology, RNA-seq revealed global insights into Hltf-regulatory networks. The most significant finding are: 1) Hltf regulates genes that encode key components of cohesin/condensin complexes that are essential for genome stability; and 2) the absence of Hltf triggers an apoptotic response. Taken together, these finding expand our understanding of Hltf signaling in development and disease.

## Supporting Information

Table S1 DNAnexus Alternative Splicing analyses quantified the usage for each exon by mapping read counts to the spliceome. Mapping coordinates from the spliceome were converted to mapping coordinates to the genome. Mappings of the same read to the same genomic location were combined, and their posterior probabilities summed. Exon quantification was performed to show the relative expression level of all known Hltf exons in brain.
(XLSX)

Table S2 DNAnexus Alternative Splicing analyses quantified the usage for each possible splice junction in control RNA-seq samples. This resulted in the comparison of different known splice products in addition to the identification of new splice products shown here as Hltf exon-skip events in brain.
(XLSX)

Table S3 RPKM values were used to identify differentially expressed genes. Genes whose expression was decreased in Hltf null mouse brain compared with controls are identified here.
(XLSX)

Table S4 RPKM values were used to identify differentially expressed genes. Genes whose expression was increased in Hltf null mouse brain compared with controls are identified here.
(XLSX)
